# A study of students’ use of restraint systems in school transportation services in primary and secondary schools in northern Iran: an observational study

**DOI:** 10.1186/s12887-021-03048-6

**Published:** 2021-12-15

**Authors:** Shahrokh Yousefzade-Chabok, Samira Azari, Leila Kouchakinejad-Eramsadati, Enayatollah Homaie Rad, Marjan Hosseinnia, Naema Khodadadi-Hassankiadeh

**Affiliations:** 1 Neuroscience Research Center, Guilan University of Medical Sciences, Rasht, Iran; 2 Guilan Road Trauma Research Center, Guilan University of Medical Sciences, Rasht, Iran; 3 Social Determinants of Health Research Center, Guilan University of Medical Sciences, Rasht, Iran; 4 School of pharmacy, Department of Clinical and Administrative Sciences, Notre Dame of Maryland University, Baltimore, Maryland USA

**Keywords:** Child restraint systems, Transportation, Accidents, Traffic

## Abstract

**Background:**

Traffic accidents are one of the leading causes of death and severe injury among child occupants of vehicles in most countries. This has led to the consideration of how to use restraint systems for students in school buses. The purpose of the present study was to determine the percentage of students’ use of restraint systems in school transportation services in 2020.

**Methods:**

In the present cross-sectional observational study, seatbelt use was assessed in 400 students in school transport vehicles using a checklist. The observation team sat at their vehicle, at the nearest location on one of the three sides of the school’s entrance: they had by manually registering the variable in the checklist. They focused on exactly the first vehicle parked next to the school entrance. There were two other observers to validate the observations. Data were analyzed by SPSS software (version 21).

**Results:**

The rate of using restraint systems was 11.3%, use of restraint systems in the Sport Utility Vehicles (SUVs) was significantly higher (*P* < 0.03), in areas with medium income (*P* < 0.009) and low income (*p* < 0.012) as well as when the students were sitting in the rear seats, using the seatbelt were significantly lower (*P* < 0.001). Seatbelt use in students was less in services driven by drivers over the age of 40 (*P* < 0.01) and more in vehicles driven by female drivers (*P* < 0.003) and newer vehicles (*p* < 0.001).

**Conclusion:**

School authorities must enforce traffic safety rules for school transportation services. These rules should be taught to drivers, families, and students. A restraint system must be mandatory for all students. School officials must equip their buses with seatbelts and employ school bus assistants to encourage wearing seatbelts and prevent students from standing.

## Background

Traffic accident injuries in children should be considered and precautions related to children’s transportation safety should be taken [1]. Severe traffic accidents are one of the 15 leading causes of death in children and the second leading cause of death between the ages of 5 and 15 [2]. In 2010, in the United States, about 1000 children under the age of 15 were killed and 171,000 injured in traffic accidents [3].

In Canada, these accidents are the cause of death and severe injuries among children under the age of 14 [4]. In the United States, more than 180,000 occupants of children under the age of 12 were involved in traffic accidents [5]. In the UAE, child deaths from traffic accidents are increasing rapidly [6] and in Iran, on average, 2 children die or are seriously injured per day. These accidents cost millions of dollars of damage every year [1]. It is believed that the death of many of these children can be prevented by the proper use of restraint systems in vehicles [7].

The anatomical body features of children are different from those of adults. This will lead to unique patterns of injury in children than in adults. An example of these features is the increase in head-to-body ratio [8]. Children should be restrained based on their age, weight, and height. Due to their physical, age, and weight characteristics, children are not allowed to use adult seatbelts and one of the necessary protective devices and alternative to seatbelts in this category is a child booster seat [1]. Child safety advisors, recommending booster seats for children over 4 years of age until they are big enough to use a seatbelt by the age of 8 to 9 years [7]. Reducing deaths and injuries from traffic accidents is highly dependent on the use of a variety of restraint systems [5]. In addition to the restraint system, the position of the child in the chair is also crucial, and the back seat is 35% safer for children than the front seat [7]. Sitting in the back seat of a vehicle in the event of an accident is a factor in preventing severe injuries [9], which increases significantly with the use of seatbelts or other restraint systems [10].

Unfortunately, only a small number of countries in the world use child safety devices in cars [1], and there is evidence that developing countries use fewer restraint systems [11].

School transportation is a special and important issue in society since it includes a very sensitive age group. Therefore creating maximum safety for students is an essential measure in any society [12]. Transportation in schools includes all methods of transferring students to schools. Walking, cycling, using private cars, buses and taxis are all modes of transportation to school,and students, parents, relatives, friends, teachers, drivers,and school bus operators, are the main stakeholders. Each of them plays an essential role and is responsible for student safety [11, 12]. Parents should adopt appropriate strategies to prevent traffic injuries, especially on the way to school, such as appropriate restraint in the car (e.g. Booster seats, seatbelts), helmets, and improved pedestrian safety and in cooperation with safety authorities enforce seatbelt and restraint rules for students [13].

In an observational study, the use of restraint systems for children under 12 years of age was about 4.3% and mostly when female drivers were driving on highways. In Iran, the use of restraint systems has been higher in higher-income families (50 million Iranian Rials per month) [14]. On the other hand, it is reported that the new model/make cars have more advanced restraints systems for children, but in most low model/make cars, such advanced restraints have are not installed for children [15].

The statistics show that a child who goes to school by car is seven times more likely to be involved in a traffic accident than a child who travels by school bus [16]. This means that school transportation by bus has a higher level of safety. In some countries, the private school system seems to be more organized than the public school transportation system. For example, private school students are being picked up from home and dropped off at schools (which seems safer), while public school students are assigned special stations for this purpose (which may be far from their place of residence). In addition, all private elementary schools offer a school bus attendant who is responsible for student safety on the bus, a service that unfortunately is not provided to public school elementary school students [12]. In a study in Nigeria, where 127 students were seen leaving a private school, despite the high level of parental awareness of restraint systems (%85), the use of restraint for students in cars was very weak and 24% was due to the unavailability of restraint systems [17].

Various factors can determine the safety of school transportation. Traffic violations are a common cause of collisions, especially seatbelt violations [18]. Parents consider several factors to be a threat to the safety of students on the school bus and are concerned about this. These include speeding, poor driving, students not wearing seatbelts, not monitoring their fastening, drivers’ lack of understanding of the school bus laws and children’s behavior [19].

School bus accidents injure child occupants and in some cases lead to their deaths, so schools are increasingly installing three-point belts on their buses to increase the safety of their students. It is necessary to pass such laws to install seatbelts on school buses. The direct benefit of adding seatbelts to school buses is to increase student protections and safety, and its indirect benefits include reducing the distraction of school bus drivers and bullying or student fights. The cost of this operation has been one of the reasons for resisting the implementation of these laws due to a reduction in the number of children transported by school buses [20].

A study refers to the indirect positive effects of seatbelts (especially 3-point / shoulder belts) on school bus safety. Such effects include improving and calming behavior on the bus and reducing driver distraction on the school bus [21]. Therefore, buying large school buses with seatbelts can have suitable and undeniable effectiveness and profitability [22]. In another study, solutions were proposed that would not require the purchase of additional buses if the law made it mandatory to equip school buses with seatbelts [23].

Improper use of restraint systems is another problem whose consequences are dangerous for children [2]. In one study, the lack or misuse of the inhibitory system was 35.5%. The ISS (Injury Severity Score) was significantly higher in the unrestrained group than in the restrained group. The unrestrained group was mostly in the ages of newborn to 7 years and the highest use of inhibition was in the ages of 17–18 years [8]. Its consequences are dangerous for children and can be corrected with education [2]. Parents should be taught that the use of restraint systems is critical to the safety of children and reduces the severity of injuries and deaths from accidents [24]. They should be aware that traffic regulations prohibit children under the age of 12 from riding in the front seat of a vehicle, as well as holding a child while driving [2].

To date, no observational study has been conducted to examine the extent of the use of restraint systems and other factors associated with the use of restraint systems for students and their drivers in the school transportation services. Student safety responsibility in school transportation services is a heavy-duty (i.e. Due to the low compliance of this age group with restraint laws, school safety experts have a heavy responsibility in school services).

Awareness of the student’s level of safety during transportation to the school and the factors associated with it helps school safety stakeholders identify and address school safety problems. This needs developing new school safety checklists and selecting prevention methods that ensure the highest degree of success.

## Methods

The present study is a cross-sectional observational study that started after approval by the ethics committee with the ID (IR.GUMS.REC.1399.182 (and obtaining written permission from the Vice Chancellor for Research of Guilan University of Medical Sciences and the director of the Road Trauma Research Center. The purpose of this study was to determine the percentage of students’ use of restraint systems in school transportation services in 2020.

### Study setting

The observed population was students and their drivers who met the inclusion criteria and sat in a vehicle with at least one student (private vehicle) to twenty students (a bus). The school transport service in the present study meant both private vehicles [(Sedan, Sport utility vehicle (SUV), taxis and call taxis, etc.] as well as public vehicles (school buses and vans, etc.). The research environment was around primary and secondary schools.

Inclusion Criteria:

1. The vehicle in which the student was riding must have the tag of the city under study.
2. Primary and secondary schools were selected.

Exclusion criteria:

1. Students who walk to school.
2. Students who go to school by motorcycle or bicycle.

### Study design

At first, the areas and regions of the city “Rasht”, are classified into three classes: high income (rich), medium-income (middle), and low income (poor) (according to the statistics of the municipality and the Statistics Center, the average score of the neighborhood index is their criterion), then from each region, 10 primary schools and 10 secondary schools were selected for viewing. The observers were three intern medical students interested in studying in the field of traffic accidents who had previously been trained at the Road Trauma Research Center to observe and complete a checklist. These areas (around the schools) were selected because:

1. Vehicles with child/adolescent student passengers (primary and secondary school) are frequently seen on these sites .2. The sampling place was safe for the observers. 3. The vehicles were stopped or had a very low speed so they were completely visible. 4. It is a place where it is possible to better observe the use of a restraint system for students and their drivers. 5. Traffic was not created during observation. 6. It was possible to see students with the age and class groups required in this study (7 to 15 years). At each site, sampling was performed during two-hour periods in the morning (7–8,30 AM) and in the evening (3,30–5 PM) and from Saturday to Wednesday when schools were open.

### Data gathering procedure

Observers parked their vehicle on the nearest empty side of the school entranced and looked inside the approaching vehicle until it came to a complete stop. When several vehicles stopped at the same time. They focused on exactly the first vehicle parked next to the school entrance. They checked the view checklist they had by manually registering the variables. There were two other observers to validate the observations. Vehicles that had frosted glass, excess occupant, or for any reason invisible cabin, were not included in the study. We instructed the observers if the student used a booster seat or any type of seatbelt (two or three points closed) as “correct” and if he did not wear any, “no restraint”, if used size-inappropriate restraints for example due to small size of the child and the need for a booster seat or loose seatbelt, instead of a seatbelt incorrectly. The student who was wearing a seatbelt would open the seatbelt when disembarking and could be identified from a student who was not wearing a seatbelt at all. We categorized drivers concerning age when the driver is young, in the age group under 25 years, if he is middle-aged, in the age group of 25–40 years, and if he is old, in the age group over 40 years. The student age is also classified as follows (all students who travel to primary schools in the age group of 7 to 12 years and all students who used to travel to secondary schools in the age group of 13 to 15 years).

### Data collection tools

#### Checklist

Observer checked the listed items according to the instructions received: School location (high, medium and low income), type of vehicle (sedan, van, school bus, taxi, call taxi, others), vehicle dating (old / new), driver sex (male/female), driver age (younger drivers in the under-25 age group, middle-aged drivers in the 25–40 age group, and older drivers in the over 40 age group.), driver seatbelt use (yes/no), student age (all students who travel to primary schools in the age group of 7 to 12 years and all students who used to travel to secondary schools in the age group of 13 to 15 years), student sex (boys/girls), type of seat (front / back seat), student position (sitting on lap / standing/ sitting), student restraint use (yes/no), restraint condition (correct, no restraint, incorrectly use of size-appropriate restraints or loose seatbelt, The small size of the child and the need for a support seat instead of a seatbelt), type of restraint (booster seat/ seat belt 2 or 3-point belts). To design the checklist, we were inspired by these three articles [7, 11, 12] and the checklist was content validity confirmed by the faculty members of the Road Trauma Research Center.

### Sample size

According to a study by Aidoo et al. (2019), the use of a restraint system was 4.5% [7]. Considering the error of 0.025 and 0.05, the sample size with the ratio calculation formula was 265 samples, which considering 20 schools, the number of samples will be equal to 365, which was increased to 400 to make sure the sample size is enough.

### Data analysis

Descriptive statistics of frequency, mean percentage, were used to examine the demographic variables of driver and student. The restraint system was divided into two categories based on the researcher’s judgment: restrained, unrestrained, and the analysis were performed accordingly. A binary logistic regression estimator was used to determine the relationship between these variables. After completing each checklist, the data were immediately entered into SPSS software version 21 and statistically analyzed.

## Results

In the present study, 400 students aged 7 to 15 years in primary and secondary school were observed in February 2017 in terms of using the restraint system. The majority, were male drivers 60% (*n* = 240), male students 53% (*n* = 212), students in the van 18.8% (*n* = 75), in the back seat 66.8% (*n* = 267), 50.3% (*n* = 201) of drivers were using seatbelts but 88.8% (*n* = 355) of the students had no restraint. Of the 45 students who used restraint systems, none used a booster seat and all had seatbelts. 85.8% (*n* = 343) of students who did not use the restraint system (or did not use it properly) were in a sitting position. 56.5% (*n* = 226) of the samples were from medium-income areas (Table 1).

**Table 1:** Demographic characteristics and restraint status of students (*n* = 400)

Characteristics	n (%)	Characteristics	n (%)
Driver Sex		Student sex	
Male	(60)240	Boy	(53)212
Female	(40)160	Girl	(47) 188
Type of car		**Driver seat belt use**	
Sedan	(60)243	Yes	(50.3) 201
SUV	(19) 4.8	No	(49.8)199
van/School bus	(75) 18.8	**Student restraint use**	
Pickup	(0.3) 1	Yes	(11.3) 45
Taxi/call taxi	(15.5) 62	No	(88.8)355
Type of seat		**Restraint condition**	
Front seat	(33.3)133	Correct	(11.3) 45
Back seat	(66.8)267	No restraint	(88.5)354
School location		Incorrectly	) 0.3(1
High income	(9.5)38	Type of restraint	
income Medium	(56.5)226	Booster seat	(0)0
income Low	(34)136	Seat belt	(100)45
Student position			
Sitting on lap	(0.3)1		
Standing	(3)12		
Sitting	(85.8)343		

Table 2 shows the role of different variables in the rate of students’ use of the restraint system using logistic regression. In the regression, the dependent variable had two values of 1 (using restraint system) and 0 (not using restraint system as the base) it clearly shows that in cases where the school service was an SUV, the rate of use of restraint systems by the student is more (OR 3.88, 95% CI 1.14–13.2) (*P* < 0.03). There is also a significant relationship between school location and the use of restraint systems so that in the central areas of the city with a medium-income level of living the rate of using restraint is more (OR 0.19, 95% CI 0.055–0.66) (*P* < 0.009) and in the southern part of the city with a low level of income the rate of restraint by the students is less likely (OR 0.2, 95% CI 0.058–0.7) (*P* < 0.012). There is also a significant relationship between examining the student’s sitting in the back and the use of restraint systems (*P* < 0.001) in which students sitting in the back were less likely to use seatbelts than those sitting in the front seat (OR 0.4, 95% CI 0.015–0.15) (Table 2).

**Table 2:** The role of different variables in students’ use of the restraint system

Variable		OR	Standard. Error	95% CI		*P*-value
				Lower Limit	Upper Limit	
Constant coefficient		0.27	0.4	0.015	4.87	0.377
Driver seat belt use(Yes) Base: No		4.5	3.73	0.88	22.91	0.069
Driver age (> 40) Other: base age (< 40)		0.58	0.39	0.15	2.19	0.42
Driver sex (Female) Base: Male		1.61	0.76	0.63	4.06	0.31
Type of car	Sedan	Base v	–	–	–	–
	SUV	3.88	2.42	1.14	13.2	0.03
Vehicle age		2.047	1.51	0.48	8.69	0.33
Student age		0.98	0.56	0.31	3.03	0.97
Student sex (Female) Base (Male)		1.57	0.89	0.51	4.79	0.42
School location	High income	Base	–	–	–	–
	Medium income	0.19	0.122	0.055	0.66	0.009
	Low income	0.2	0.128	0.058	0.7	0.012
Student position (Back) Base(Front)		0.4	0.028	0.015	0.15	0.001>

Table 3 shows the role of different variables in the driver’s use of seatbelts. Based on the results, students in the school services driven by drivers more than 40 years old were less likely to use seatbelts compared to those students in the services whose driver was younger (less than 40 years), and it was significant (OR 0.03, 95% CI 0.002–0.48), (*P* < 0.01). On the other hand, the odds of using seatbelts by students in school services driven by female drivers was more compared to those services conducted by a male driver (OR 2.54, 95% CI 1.36–4.7), (*P* < 0.003). There was a significant relationship between the type of vehicle and the use of seatbelts, so that the odds of seatbelt use was much less in taxi and call taxi drivers (OR 0.13, 95% CI 0.04–0.35) (*p* < 0.001), as well as vans and mini-busses, than other vehicles (OR 0.10, 95% CI 0.04–0.26) (*p* < 0.001). Another important and significant variable was the age of the vehicle, in which students sitting in newer vehicles, were more likely to use seatbelts compared to those sitting in older vehicles (OR 4.4, 95% CI 2.3–8.1) (p < 0.001) (Table 3).

**Table 3:** The role of different variables in the driver’s use of seat belts

Variable		OR	Standard. Error	95% CI		***P-value***
				Lower limit	Upper limit	
Constant coefficient		1.32	1.2	0.22	7.8	0.75
Age	25<	Base	–	–	–	–
	25–40	0.35	0.29	0.06	1.86	0.21
	40 >	0.03	0.04	0.002	0.48	0.01
Driver sex- female (base: male)		2.54	0.81	1.36	4.7	0.003
Type of vehicle	Sedan	Base	–	–	–	–
	Van/school bus	0.10	0.04	0.04	0.26	0.001>
	/Taxi/call taxi	0.13	0.06	0.04	0.35	0.001>
Vehicle age (new) Base (old)		4.4	1.38	2.3	8.1	0.001>

## Discussion

This study, discovered some new factors affecting the use of restraint in students and seatbelts for drivers besides demonstrating the level of seatbelt use in school transportation services for students, in general.

In the present study, 33.3% of students were sitting in the front seat. Approximately similar to a study conducted in Kumasi, Ghana, in which 26% of children sat in the front seat [7]. Studies have shown that the rate of children sitting in the front seat has been in the range of 12 to 50% [25] and the results of the present study showed that in northern Iran, the rate of students sitting in the back seat was consistent with the results of some similar studies, but still much lower than in developed countries. The study also shows that several students were standing in the school service. In one study, high-risk student behaviors were observed on school buses, including standing on a moving bus and standing before the bus stopped completely.

These high-risk behaviors were significantly higher in primary school students than in secondary school students and the afternoon when returning home. In many developed countries, in school transportation safety guidelines from student codes of conduct, a “no standing” rule; to school bus drivers; It is taught alongside other safety rules [18].

In the present study, only 11.3% of students used seatbelts as a protective method. In different studies, this rate varied from 6% [25], 16.7% [6] and 54.2% [26]. In other studies, 60% [10] to 92% [27] of children used a restraint method. In one study, the majority of teens who died in accidents, did not use seatbelts, and even a small increase in seatbelt use could be decisive [28]. Of course, the age group of children in different studies is not the same for example, in studies where children were younger and traveled by private vehicles, and they were more under the supervision of their parents, were more restrained. Less attention is paid to restraint for older children.

On the other hand, when parental supervision is reduced, as, in the case of students in the present study, the use of student restraint is less. However, in the north of Iran, with such a high rate of motor vehicle accidents [29], we had one of the lowest rates of use of seatbelts among students. Therefore, the need for parental education is palpable. Although in one study, the majority of parents (82%) believed that restraint protects their children in the event of a car accident, only 47% of parents always used restraint for their children [30].

The study shows that the use of a booster seat was zero. Similarly, in the study of Pan et al. (2011) in Shanghai, the use of a booster seat was only 2.2% [25]. It is necessary to use a booster seat for children and fatality analysis for the period 2008–2016 has reported effective support for children aged 6–9. To increase the use of booster seats, the National Highway Traffic Safety Administration encourages state legislators to enforce booster seat laws [31]. It seems that due to not using a booster seat in the present study, there is a need to educate and introduce the booster seat to the parents of students and encourage them to use it.

Another finding of this study was that about half of the students’ drivers wore seatbelts. Similarly in the study of Porter et al. 52.1% [2] and the study of Oxley et al. (2018), 44.8% of drivers wore seatbelts [11]. It is noteworthy that the use of seatbelts in drivers has been significantly higher than passenger students, which seems to be related to the fact that in the north of Iran, drivers will be fined if they do not wear seatbelts, but no fines for sitting in the rear seats without seatbelts fasten. As previously reported, increased law enforcement pressure on driver’s leads to better compliance with driver seatbelt laws [32], and better compliance with traffic laws is associated with reduced traffic deaths [33].

In the present study, in cases where the school service was a SUV, the use of seatbelts for students was higher. Also, in the student living in the affluent neighborhood, seatbelt use was more common. In an another study, similarly, the use of restraint systems for children was directly related to income and financial living status, so that in high-income people this rate was much higher than in low-income families [34].

Equipped with a working seatbelt, which is provided in newer make/model and more expensive cars and for children living in affluent areas, seems to have contributed to the increased use of seatbelts by these students, as previously reported new cars to have more advanced restraints for children, but older cars with lower make/model, do not have such advanced restraints for children [15]. Also, when using this type of car, the parents’ supervision over the use of restraint by the student is direct, but in school buses, the supervision is with the driver only.

In the present study, students in the rear seat used much fewer seatbelts than those in the front seat. Similarly, in a study by Oxley, et al., Children in the front seat used seatbelts more than those in the rear seat [11]. It has previously been reported that in developing countries, most cars do not have seatbelts in the rear seats, and it has been suggested that local car repairmen set up a business to produce low-cost seat belts so that children can have appropriate age restraint and also children should be encouraged to wear a seatbelt in the rear seat [15]. In addition to the lack of fines, which is a reason for lower seatbelts to be worn in the rear seat [32], school transportation drivers believe that the student has a safer place in the rear seats and they need less restraint.

According to the results, the older the drivers (over the age of 40), the lower the seatbelt use. Similarly, in a study by Okamura and colleagues in Japan, drivers 50 years and older were less likely to wear seatbelts. Also, child occupants of these vehicles used fewer restraint systems [35]. Accompanying musculoskeletal problems sometimes force older drivers not to wear seatbelts [36]. However, in one study in Egypt, young drivers wore seatbelts less than middle-aged drivers, and older drivers showed more careful driving behaviors, including fewer errors and violations [37]. In a study in Iran, in 63% of the provinces, the least adherence to seatbelts was in young people [38]. However, there is a need to carefully examine the age of drivers in future studies to determine why school service drivers wear fewer seatbelts when they are older. Possibly, being on the school bus or service itself is a factor contributing to not following the rules of wearing a seatbelt in this group.

The study shows that female drivers wore seatbelts more than male drivers, but there was no relationship between students’ seatbelts and driver gender. Similarly, in other studies, seatbelts were used more by female drivers, and in the same cars, children also used more restraint systems [39]. Porter et al. in Turkey found that female drivers were more likely to wear seatbelts for their child occupants in the car, and the number of children being held in the arms of another occupant was higher in the presence of the female driver [2]. In one study, seatbelts were fastened by occupants depending on the driver’s gender, and if both were the same, if the driver did not fasten, the occupant would wear fewer seatbelts [40]. In the present study, women seem to be different from men in terms of attitude, behavior, and risk perception. As previously reported, women are different from men in terms of the level of concern and are more inclined to be careful in traffic accidents to prevent their consequences [30]. Therefore, it seems that the reason why female school bus drivers in our study followed the seatbelt rule more, follows the same rule that female drivers are more cautious.

In this study, the use of seatbelts in taxi and call taxi drivers, as well as vans and buses was much less than other school transportation services (private sedan and SUV). In a similar study, the type of vehicle showed a significant relationship with the use of child restraint. Also, child occupants in SUV cars had the most and in the cabs, had the least use of restraint systems. In a study, the use of child restraint ensured the use of seatbelts in the driver [39]. In another study, the occupants of private cars used seatbelts less than rental cars [41].

In the present study, drivers of older vehicles wore seatbelts significantly less than newer vehicles. However, the age of the vehicle had no significant relationship with the student’s use of the seatbelt. In one study, it was reported that in low- and middle-income countries, even newer cars do not have enough protection for children sitting in the rear seat [15]. Therefore, it is possible that in this study also, older cars were less equipped with restraint systems for drivers than newer cars.

One of the limitations of the present study was that the reporting of cases that did not use correct restraint was very low. It is probably due to the study method that the observation was anonymous. If the study method is changed to simultaneous observation and interview, then it will be possible to observe more closely and distinguish between correct and incorrect restraint, but in that case there was a possibility of losing a lot of useful information, including students standing in the service.

## Conclusion

School authorities must enforce traffic safety rules for school buses. School officials must teach these rules to drivers, families, and students. Seatbelts should be mandatory for all students, whether those traveling by public transport or those using private vehicles. They should wear seatbelts when all students get on and off and monitor them. School officials should equip old buses with seatbelts or replace them with new ones, and if possible, place a school bus assistant next to the driver on the school bus to prevent students from standing and to monitor they are wearing a seatbelt.

## Data Availability

The datasets used and/or analyzed during the current study available from the corresponding author on reasonable request.

## Abbreviations

UAE: United Arab Emirates
ISS: Injury Severity Score
SUV: Sport Utility Vehicles
